# Supplementary material for: Emotions, perceived threat, prejudice, and attitudes towards helping Ukrainian, Syrian, and Somali asylum seekers
Source: PLoS One. 2023 Sep 13;18(9):e0290335. doi: 10.1371/journal.pone.0290335 (PMC10499223; doi:10.1371/journal.pone.0290335)
Supplement: Attachment — Submitted filename: Response to reviewers.docx [file pone.0290335.s002.docx]

**Emotions, perceived threat, prejudice, and attitude towards helping Ukrainian, Syrian, and Somali asylum seekers** – Response to reviewers

**Journal requirements**

1. Style : Changed, in line with the templates sent to me.
2. Additional details re participant consent: *Participants marked their consent on the online survey, and those who selected ‘no’ to any of the consenting questions were unable to complete the survey, and were simply thanked for their time.*
3. Data availability: Data set uploaded

**Reviewers’ comments:**

1. Writing style: The paper has been reviewed by a very experienced researcher colleague, for whom English is a first language. Changes to aid readability have been made throughout, with the reordering of some arguments to enhance clarity.
2. Prejudice and stereotypes: Inserted more literature about prejudice, and highlighted the link to stereotypes.

*Stereotypes are underlying beliefs about a group of people, and attributions of specific characteristic to them; they can be positive or negative [44,45]. Some research has found that stereotypes about immigrants tend to be ambivalent, for example low warmth and high competence, or high warmth and low competence [46]. Refugees as a category are generally perceived to be low on both warmth and competence, though the background of the refugees has some impact on perception of warmth [47]. Stereotypes can lead to prejudice, and are therefore key to our understanding of this phenomenon.*

*Prejudice tends to be a devaluation of people because of their group membership, leading to potential harm or negative consequences [48]. It relates to a judgement made before, or irrespective of, actually meeting the person. For instance, dark-skinned young people in Australia have been perceived as religiously-indoctrinated terrorists simply because of their appearance [44,49]. Prejudice is a set of hostile attitudes or feelings towards someone [44], following an evaluation of the group the person belongs to [45]. It is both irrational, in that prejudice persists in the face of evidence to the contrary, and negative [44]. Prejudice can be seen as an evaluation that includes both cognitive and affective responses [29], and often reflects a perception of the other person as low on warmth and competence [50].*

3. The use of “Warmth” as a label for one of the factors is confusing in relation to Warmth and Competence as identified by Fiske: Relabelled the factors Warmth and Contempt to Positive Emotions and Negative Emotions.
4. “These claims seem exaggerated, even considering the literature that is referenced in this paper”, expressed with regard to material in page 3 and pages 7-8: Reviewed further literature, focussing on material suggested by Reviewer 1, and integrated this material into the paper.

What was previously page 3: *There is agreement in the current research literature that perceived threat influences cognitive evaluations, such as prejudice, and negative emotions towards people [15,16]. In addition, empirical studies have demonstrated that prejudice and negative emotions increase restrictive attitudes towards refugees and reluctance to help them [17,18]. However there is a gap in the literature concerning how emotions, perceived threat, and prejudice may come together to influence attitudes towards helping different groups of asylum seekers. Research has shown that refugees, asylum seekers, undocumented migrants or other incomers who are Muslim or not White are seen as a drain on a country, in comparison to generally-White migrants (such as Germans, Canadians or British) who are perceived as assets [19]. However, it is still unclear whether European citizens distinguish between asylum seekers from Europe and those from other parts of the world. This paper presents a study of Europeans’ perceptions of asylum seekers from Ukraine, Syria, and Somalia. The research investigated possible differences in the negative emotions, perceived threat, prejudice and attitudes that Europeans hold towards people arriving from each of these countries. In addition, the theoretical understanding of the relationship between these variables was statistically examined, to explore how they fit together and inform each other.*

What was previously pages 7-8: *There is empirical evidence that perceived threat, emotions, and prejudice have an effect on attitudes towards helping refugees or asylum seekers. The inter-relationship between these factors has been measured in relation to outgroups. For example, perceived threat predicts emotions and behavioural intentions, and emotions predict behaviour intentions [61]. However participants in that study were not asked to consider asylum seekers. In addition, it is argued that different outgroups are perceived as posing different types of threat, also depending on the ingroups’ context [62]. While there are indications that European participants would look more favourably upon Ukrainian asylum seekers, as compared to Middle Eastern or African ones, empirical evidence is currently lacking.*

5. Table 4: Kept only the values above the diagonal (Reviewer 2’s suggestion).
6. 2-way ANOVA: Results are now reported

*In terms of participants’ country (see Table 2), there were significant main effects for negative emotions (F(1,281)=6.05, p<.05, η_p_^2^=.02) and perceived threat (F(1,281)=4.60, p<.05, η_p_^2^=.02). That is, participants in Malta reported higher ratings on negative emotions and conditional prejudice, and lower ratings on positive emotions and attitudes towards helping, than participants in the UK. With regard to asylum seekers’ country of origin, there were significant main effects for negative emotions (F(2,281)=14.05, p<.001, η_p_^2^=.09), positive emotions (F(2,281)=20.39, p<.001, η_p_^2^=.13), conditional prejudice (F(2,281)=3.87, p<.05, η_p_^2^=.03), and attitudes towards helping (F(2,281)=3.295, p<.05, η_p_^2^=.02). None of the interaction effects were significant.*

7. Mediation analysis: Reviewer 1 asked to add to the limitations section some of the concerns about this type of analysis. Done – see below.

*Finally, in terms of the mediation analysis, evidencing mediation is challenging [73], and the mediation analysis in this study has its weaknesses. It is acknowledged that caution should be used when inferring mediation from cross-sectional data, as this type of data collection does not account for the temporal element of the experience of variables [74]. That is, a cross-section mediation analysis may yield different results when compared to a longitudinal study examining the same variables.*

In the Results section, I now generally refer to indirect effects, rather than mediation. There are two references to mediating variables, simply because it was difficult to reword ‘mediating variable, in a mediation analysis, without the language becoming cumbersome, e.g. *Hence negative emotions was a significant mediating variable in these relationships, with the indirect effects being larger than the direct effects*. There are also three references to prejudice as a mediating variable in the Discussion, for this same reason. If the editor still believes that this should be amended, I will make sure to do this. The wording in the Abstract has been amended to avoid ‘mediating variable’.

8. Acknowledging some of the sociostructural variables: This has now been acknowledged.

*In addition, there is evidence that ethnic groups from lower latitudes (closer to the equator) are perceived as less trustworthy, competent, and capable, when compared to people from higher latitudes [37]*

9. Tying final discussion more closely to the theoretical concepts presented in the introduction: This has been done, and the below is some of the new material, which is spread across various sections of the Discussion.

*The literature about intergroup threat identifies two types of threat: realistic and symbolic [25]. The confirmatory factor analysis in the current study questions this distinction. Looking at media and political discourses, politicians may emphasise symbolic threat, with media focussing more on realistic threats [71]. Possibly the various discourses lead people to experience threat more ‘holistically’, rather than in its theoretical subtypes. Further study could examine how people experience these theoretical sub-types of threat.*

*The finding that both emotions and perceived threat have an effect on prejudice confirms the understanding of prejudice as having both an affective and a cognitive element [29].*

*Recognising that people evaluate asylum seekers as more or less deserving of help [57], we can now understand further how these assessments are made. While perceived threat does influence intentions to help [60], and is associated with more negative attitudes [15,31], this involves a further evaluation of asylum seekers. Hence there is a two-step evaluation: level of threat, and negative attitudes (prejudice).*

*Both positive and negative emotions had a stronger relationship with classical prejudice than with conditional prejudice. With classical prejudice being more overt or blatant [54,55], this suggests that conditional prejudice is possibly not simply a more acceptable way to articulate prejudicial evaluations, but perhaps a less strong or less negative bias. That is, the distinction might be more of experience than of expression.*

*The indirect effect of perceived threat on attitudes towards helping (via prejudice) was stronger than that of emotions on attitudes towards helping. This indicates a stronger cognitive than emotional, effect on attitudes towards helping. In other words, what people think about asylum seekers and possibly how they perceive them, and the implications of accepting them, has a stronger effect than their feelings about asylum seekers. However, unsurprisingly, it may be difficult to separate the different influences. There is agreement in the current research literature that perceived threat influences negative attitudes and emotions towards refugees [15,16]. Perhaps because of indications that cognition and emotions interrelate [72], it is necessary to focus on how they work together to influence attitudes towards asylum seekers. This is an outstanding point of interest which requires further investigation.*

10. Considering benefits of asylum seekers: This has been added as a limitation/area for further research.

*As recent studies are doing [28], it would also be helpful to explore how people from host countries think refugees may bring benefits, delving into potential impact of asylum seekers’ country of origin with regard to this.*
